# Genetic data normalization for genomic medicine: a Fast Healthcare Interoperability Resources Genomics reference implementation

**DOI:** 10.1093/jamia/ocaf136

**Published:** 2025-09-11

**Authors:** Robert H Dolin, Nicolae-Mihai Todor, James Shalaby, Huda Arsalan, Eshani Shah, Nedah Basravi, Ammar Husami, Akash Rampersad, Bret S E Heale, Srikar Chamala

**Affiliations:** Elimu Informatics, El Cerrito, CA 94530, United States; Independent Data Lab, Dublin D15 CD79, Ireland; Elimu Informatics, El Cerrito, CA 94530, United States; Elimu Informatics, El Cerrito, CA 94530, United States; Luddy School of Informatics, Computing, and Engineering, Indiana University, Indianapolis, IN 46202, United States; College of Pharmacy, Touro University California, Vallejo, CA 94589, United States; Division of Human Genetics, Cincinnati Children’s Hospital Medical Center, Cincinnati, OH 45229, United States; Department of Pediatrics, University of Cincinnati College of Medicine, Cincinnati, OH 45219, United States; Oak Bioinformatics, Fairfax, VA 22030, United States; Humanized Health Consulting, Salt Lake City, UT 84102, United States; Keck School of Medicine, Department of Pathology, University of Southern California, Los Angeles, CA 90033, United States; Department of Pathology and Laboratory Medicine, Children’s Hospital Los Angeles, Los Angeles, CA 90027, United States

**Keywords:** genomics, genetic data normalization, variant annotation, FHIR genomics, precision medicine

## Abstract

**Objectives:**

Demonstrate the ability to encapsulate clinical-grade genomics data normalization algorithms within a FHIR Genomics reference implementation.

**Background:**

Variability in genomics data representation is a significant impediment to precise search, clinical decision support rule writing, variant annotation, and more. Such variability is problematic not just for genetic variants, but also applies to HLA alleles, phenotype codes, and more. Here, we provide an overview of genomic data variability and normalization algorithms, focusing on three key areas: genetic variants, HLA alleles, condition and medication variant annotations. We describe and demonstrate the strategies used in a public open source FHIR Genomics reference implementation.

**Materials and Methods:**

We developed a set of design considerations, which we used to weigh different normalization approaches. All data (ingested patient data, ingested knowledge, query parameters) are subjected to normalization. Variant normalization leverages the biocommons/hgvs python package. HLA allele normalization leverages the py-ard python package. For variant annotation terminology variability (for conditions and medications), we leveraged FHIR-based ConceptMaps.

**Results:**

Algorithms for normalization of genetic variants and HLA alleles, and terminology translations, have been implemented and deployed in a public open source FHIR Genomics Operations reference implementation. All data and source code described in this report are located at https://github.com/FHIR/genomics-operations, and deployed at https://fhir-gen-ops.herokuapp.com/. Every normalization strategy examined to date has known limitations.

**Conclusion:**

While we report on our experience successfully encapsulating genomic data normalization in FHIR Genomics Operations, the challenges and solutions identified are broadly applicable to many other contexts.

## Objectives

Genomic data can be represented in many, often synonymous, ways due to multiple standards, redundant representations within a standard, and differences in testing methodologies that lead to differences in data granularity. Variability in data representation is a known barrier to matching a patient’s genetic profile against knowledge—for clinical trials matching, for prediction of genetic predisposition to disease, for precision medication selection, for genetic risk calculation, and more.

Here, we sought to assess and demonstrate the ability to encapsulate data normalization within clinical-grade standardized Fast Healthcare Interoperability Resources (FHIR)-based genomic application programming interfaces (APIs, also known as “FHIR Genomics Operations”),^1^^,^^2^ shielding clients from complex bioinformatics calculations, in an attempt to simplify downstream tasks for researchers, clinicians, clinical informaticists, and clinical application developers.

## Background and significance

Variability in genomics data representation is a significant impediment to precise search, clinical decision support (CDS) rule writing, variant annotation, and more. As noted by Holmes et al,^3^ analysis of genetic data “can be challenging because any variant can be described using different formats, different reference sequence types and different assembly versions”. Challenges with genomic data variability extend beyond genomic variants, and include other aspects largely unique to genomics (such as variability in human leukocyte antigen, HLA, allele names) and those challenges also seen in other clinical informatics domains (such as different terminologies used by different knowledge bases).

To advance our objectives, we focused on three types of genetic data—variants, HLA alleles, and variant annotations—as these areas represent a wide range of scenarios for proof-of-concept design. For each of these areas, we provide a brief non-exhaustive overview of the types of data variability and normalization algorithms, from which we then move into a detailed discussion of our approach and how we encapsulated normalization algorithms into FHIR-based genomic APIs in a public open-source FHIR Genomics reference implementation.

### Overview of genomic data variability

#### Variants

Holmes et al^3^ provides a detailed description of variant representation standards and normalization tools. Not only are there multiple formats for variant representation but there are also multiple ways of representing a variant within each of these formats, as shown in Table 1.

**Table 1.** Examples of variability in the representation of genetic variants.

**Variant represented synonymously in HGVS and SPDI format**	
Variant in HGVS format	NC_000019.9:g.11200236G>A
Same variant in SPDI format	NC_000019.9:11200235:G:A
Variant represented synonymously as a difference against different reference sequences	
Variant as a change in the NM_001195798.2 transcript	NM_001195798.2:c.12G>A
Same variant as a change in the NM_001195803.2 transcript	NM_001195803.2:c.12G>A
Same variant as a chromosomal change	NC_000019.9:g.11200236G>A
Same variant as a gene change	NG_009060.1:g.5180G>A
Variant represented synonymously as a difference against different versions of a reference sequence	
Variant as a change from a GRCh37 reference	NC_000019.9:g.11200236G>A
Same variant as a change from a GRCh38 reference	NC_000019.10:g.11089560G>A
Variant represented synonymously using different justifications** (In the following example, reference sequence NC_000007.14 has a string of seven “T” bases starting at position 117548628, and we illustrate a single base pair deletion)**	
Variant expressed as a left justified change	NC_000007.14:117548628:T:
Same variant expressed as a right justified change	NC_000007.14:117548634:T:
Same variant expressed as a fully justified change	NC_000007.14:117548628:TTTTTTT:TTTTTT

Variant formats include the Variant Call Format (VCF^4^), Human Genome Variant Society (HGVS^5^) Nomenclature, Global Alliance for Genomics and Health Variant Representation Specification (GA4GH VRS^6^), FHIR Genomics,^2^ National Center for Biotechnology Information Sequence Position Deletion Insertion format (NCBI SPDI^3^), and other less common formalisms.

The notion of “variant” indicates a difference between, say, a patient sample and a reference. Where a patient’s sample differs, we say the patient has a variant at that position. But many reference sequences and reference sequence types exist, and different labs may report variants against different reference sequences.

Reference sequences are versioned (eg, NC_000019.9 is an older version whereas NC_000019.10 is a newer version of the NCBI chromosome 19 reference), and different labs may report variants against different versions of a reference sequence. Reference sequences are grouped into “assemblies,” with those reference sequences within an assembly having been developed in parallel over the same time period. Labs generally use reference sequences from assembly “GRCh37” (often referred to as “build 37” or “hg19”), developed in 2009; or assembly “GRCh38” (often referred to as “build 38”), developed in 2013.

A more subtle representational variation has to do with “justification.” In many cases, such as where a reference sequence has a range comprised of a string of repeating nucleotide bases (eg, “AAA”) and the patient has a single base deletion in that range, it generally is not known which of the repeating bases was deleted. A “left justified” representation assumes that the first base was deleted, a “right justified” representation assumes that the last base was deleted, and a “fully justified” representation assumes that one of the bases was deleted.

A large number of high quality open source tools support variant normalization, many of which are summarized and compared by Holmes.^3^ Tools for converting between formats include vcf2fhir (converts VCF to FHIR),^7^ and NCBI SPDI services (interconverts HGVS, SPDI, GA4GH VRS, VCF).^3^ Tools for converting between different reference sequences include NCBI SPDI services and the python biocommons/hgvs library.^8^ The Griffith Lab provides a nice introduction to a variety of liftover tools,^9^ and the python pyliftover library provides a suite of solutions for conversion between versions.^10^ Additional strategies for data normalization include the use of synonym databases, such as the ClinGen allele registry.^11^

#### HLA alleles

Use cases driving our implementation relate primarily to the fields of transplantation, immunology, and oncology. Transplantation scenarios generally revolve around finding a suitable donor. Where the donor or recipient have pre-existing HLA antibodies, it can be important to ensure the other party does not have the corresponding HLA allele or epitope. Immunology scenarios primarily relate to autoimmunity and correlating phenotypes with HLA alleles. Oncology scenarios are emerging and include, for instance, HLA typing to guide the use of immunotherapy.^12^

The HLA region on chromosome 6 is one of the most complex regions of the human genome, given its enormous diversity, evolution in sequencing technology (and corresponding nomenclature), differences in reporting granularity (eg, very fine grained for bone marrow transplant, less finely grained for solid organ transplant), and the presence of many “pseudogenes” (near copies of the HLA genes that are non-functional) that result in ambiguity in allele calling. As a result of this complexity, HLA findings can be reported in a number of ways, such as:

- **Serology**: eg, HLA-A2
- **Differing specificity**: eg, HLA-A*02 (one field specificity) vs HLA-A*02:101 (two field specificity) vs HLA-A*02:101:01 (three field specificity)
- **G Groups** ^13^: eg, A*01:01:01G
- **P Groups** ^13^: eg, A*01:01P
- **CWD String**: eg, B*15:01:01/B*15:01:03/B*15:04/B*15:07/B*15:26N/B*15:27
- **GL String Code** ^14^: eg, hla#3.25.0#HLA-A*01:01:01:01/HLA-A*01:02+HLA-A*24:02:01:01
- **MAC codes** ^15^: eg, HLA-DRB1*13: AKBAR
- **Epitopes** ^16^: eg, Bw4
- **Coded LOINC observations**: eg, 79712-6 (HLA-A*31:01); 49555-6 (HLA-A+B+C Ag); 79711-8 (HLA-B*58:01)

While the IPD-IMGT/HLA Database^13^^,^^17^ is the official source of HLA allele names, the complexity of HLA sequencing has led to a number of shorthand representations that encapsulate ambiguity or complexity in observed alleles. For instance, G Groups are groupings of HLA alleles that contain the same nucleotide sequences across the exons encoding the antigen recognition domain (“ARD”). HLA alleles A*01:23:01 and A*01:23:02 are both members of G Group A*01:23:01G. P Groups are groupings of HLA alleles that have the same amino acid sequence across the ARD. HLA alleles A*01:69:01, A*01:69:02, and A*01:69:03 are members of P Group A*01:69P. CWD and GL String representations have internal syntax that support reporting ambiguities—eg, the “/” in “B*15:01:01/B*15:01:03” indicates an OR-group, where one of the HLA alleles, B*15:01:01 or B*15:01:03, is present but the specific test was unable to further differentiate. Where there is significant ambiguity and where the list of possible HLA alleles present in a person is long, the list can be collapsed into a MAC code. For instance, the MAC code “DRB1*13: AKBAR” is a compressed representation of “DRB1*13:02/DRB1*13:36/DRB1*13:67/DRB1*13:96/DRB1*13:109.” HLA epitope groupings are groups of HLA alleles that contain common antibody binding sites. For instance, HLA B5, B13, and A2403 all share the Bw4 epitope. Coded LOINC observations are prevalent in clinical scenarios. Where applicable, the LOINC committee maps each LOINC code to its parts such that one can determine, for instance, that LOINC code 79712-6 is reporting on the presence of HLA-A*31:01 in blood.^18^

While various tools exist for manipulating HLA representations, the most robust tool we have identified to date is the Python py-ard library, developed by the National Marrow Donor Program (NMDP).^19^^,^^20^ This “swiss army knife of HLA nomenclature” leverages the IPD-IMGT/HLA Database to enable translations across various HLA representations, managing variation in historical resolutions and groupings.

#### Annotations

Variability exists in the representation of variant annotations. SnpEff,^21^^,^^22^ Ensembl’s Variant Effect Predictor,^23^ and Annovar^24^ can add similarly structured annotations into a VCF file, while the GA4GH Variant Annotation Specification (GA4GH VA^25^) and the FHIR Genomics standard specify exchange standards for annotation information. But in all of these cases, there remains considerable variability in terminology (eg, codes used to represent conditions or treatments). Terminology issues arise, for instance, where code systems such as SNOMED and RxNorm that are prevalent in electronic health records (EHRs) differ from code systems used to encode phenotypes and medications in knowledge bases, or where different implementations opt to use pre- vs post-coordinated codes (eg, SNOMED 1259754003 | Primary squamous non-small cell lung cancer vs SNOMED 372087000 | Primary malignant neoplasm + ICD-O-3 8070.3 | Squamous cell carcinoma).

FHIR Genomics Operations phenotype API calls can include coded conditions and/or treatments as search parameters. Where these codes differ from the native codes used by a given knowledge base, annotations can fail to be retrieved. For instance, ClinVar^26^ encodes conditions with Medgen (https://www.ncbi.nlm.nih.gov/medgen/); Clinical Interpretation of Variants in Cancer (CIViC^27^) encodes conditions with Disease Ontology (https://disease-ontology.org/) and medications with National Cancer Institute Thesaurus (NCIt); Pharmacogenomics Knowledge Base (PharmGKB^28^) encodes medications with RxNorm (https://www.nlm.nih.gov/research/umls/rxnorm/).

Terminology challenges are not unique to genomics, and have been well described (eg, Ref.^29^). Complexities relate to the fact that codes in different code systems have different granularities (eg, SNOMED's “Acoustic neuroma of left vestibular nerve” vs DO's “Acoustic Neuroma”), but may also have different meanings and/or may be hierarchically organized using different heuristics (eg, organized by body site vs organized by morphology). As a result, exact matches are much less common than broader to narrower matches, and a code in one code system may map to more than one code in another code system.

**Figure 1.**
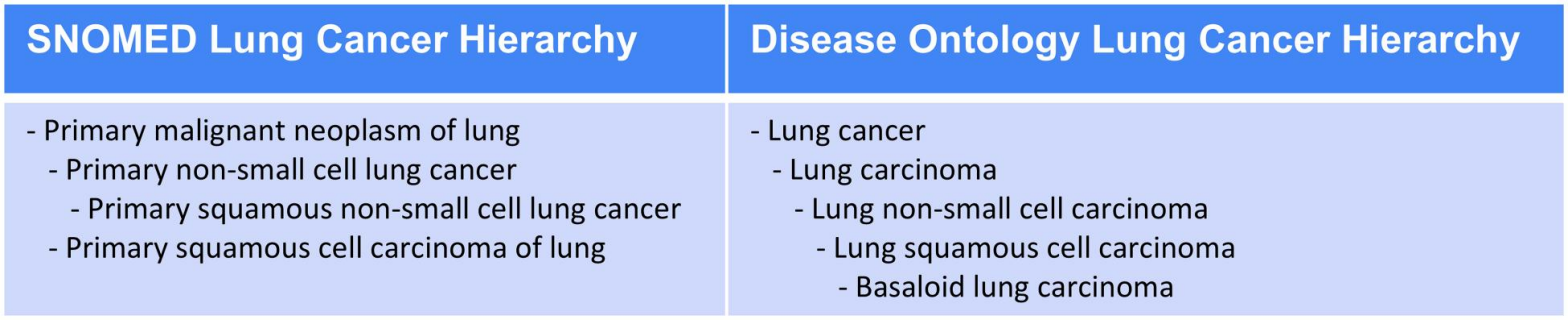
Representative lung cancer terminology from SNOMED and Disease Ontology.

To better illustrate, consider the following two hierarchies, from SNOMED and DO, shown in Figure 1, and consider the case of a client seeking therapeutic implication annotations for a patient having a SNOMED-coded diagnosis of *1259754003 | Primary squamous non-small cell lung cancer*. An exact match is DO's *DOID : 3907 | Lung squamous cell carcinoma* but a client may want to also identify knowledge associated with descendant *DOID : 7045 | Basaloid lung carcinoma* and/or ancestor *DOID : 3908 | Lung non-small cell carcinoma*.

Terminology mappings are prevalent, and vary widely with respect to supported use cases and quality. Many healthcare organizations are now formalizing terminology maps in FHIR format, making them accessible in real time via FHIR terminology services.^30^

## Materials and methods

This study uses synthetic and/or publicly available and anonymized patient data. As such, no Institutional Review Board approval was sought. Our objective for the work reported here was to assess and demonstrate the ability to incorporate robust normalization algorithms into clinical query pipelines so that they are leveraged seamlessly (eg, by clinical applications), behind the scenes. To do this, we enhanced a public open-source implementation of FHIR Genomics Operations.^1^^,^^31^^,^^32^

### Design considerations

To assess and demonstrate the ability to encapsulate data normalization within FHIR Genomics Operations, we began by establishing a set of design considerations, shown in Table 2.

**Table 2.** Design considerations for encapsulating data normalization within FHIR Genomics Operations.

Design consideration	Notes
Solution must support a wide range of scenarios.	We chose to test our strategy against 3 types of genetic data—variants, HLA alleles, and variant annotations.
Solution should leverage existing algorithms and software libraries, particularly those that have been rigorously tested, and those that facilitate maintenance (eg, an HLA normalization library that has an active development community and provides a solution for IPD-IMGT/HLA database updates).	Our goal here was not to create new normalization algorithms, but to show that many existing algorithms can be seamlessly encapsulated.
Solution should enhance rather than replace basic search capabilities.	FHIR Genomics Operations define expected behavior for “codesystem|code” and “token” based search, with the former used for exact matching and the latter generally used for case-insensitive search through a concept’s code, code system, and display. Servers may optionally broaden the use of a token to also perform close to exact searching. Normalization logic enables additional search precision.
Variant normalization strategy should resolve each variant into a chromosomal canonical form so as to enable uniform range-based search.	Normalization via conversion into a canonical form enables a client to search against that canonical form. Range-based searching is particularly useful for genetic variant data.
Solution needs to be able to run locally or on a server without the need for external API calls.	Solutions that require external API calls for normalization are generally less performant and may fail if the external service experiences downtime.
Solution’s data normalization pipeline can be applied to ingested patient data, ingested knowledge, and search parameters.	Not only patient data, but also knowledge and query parameters may need to be normalized.
Any encapsulated solution should also be able to serve as a stand-alone API utility.	In some cases (eg, with terminology translation) there are many possible scenarios (eg, one user wants to search for the best match whereas another user wants the best match and all descendants). We expose normalization utilities that give the client more options.
The choice of canonical form is based on use case analysis.	Data normalization will not necessarily yield 100% search precision, and different normal forms may have different strengths and weaknesses. Where other canonical forms exist, particularly where they may yield greater precision in certain scenarios, those other forms can be surfaced via normalization utilities.

### General approach

**Figure 2.**
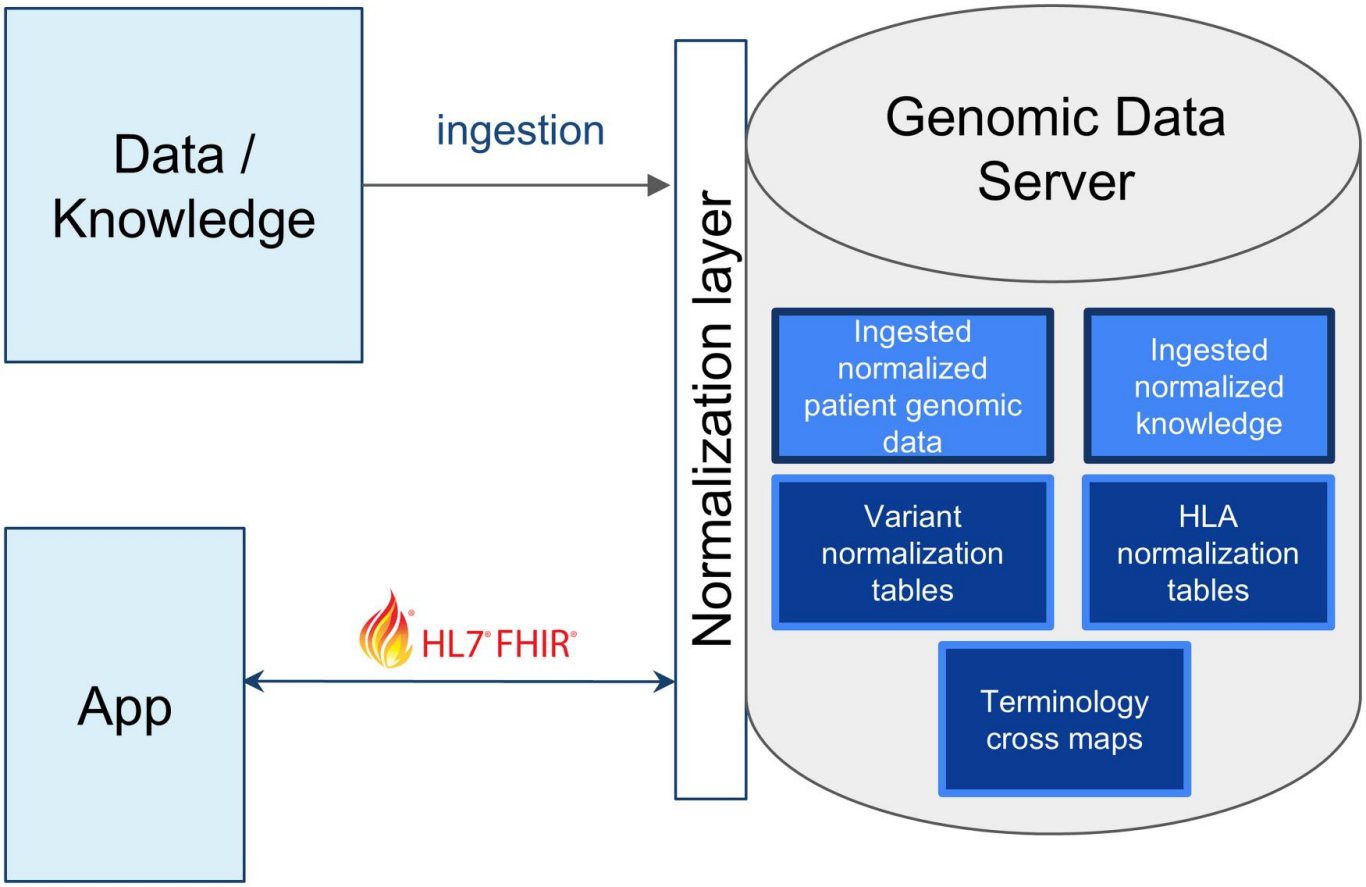
High level data normalization architecture in FHIR Genomics Operations reference implementation. The FHIR Genomics Operations reference implementation normalizes knowledge and patient data (variants, HLA alleles) upon ingestion. Apps (eg, SMART-on-FHIR software applications) communicate with the server via FHIR Genomics Operations. Many Operations contain query parameters “variants,” “haplotypes,” “treatments,” “conditions” that are normalized upon receipt by the server.

To achieve our objective of incorporating robust normalization algorithms into clinical query pipelines so that they are leveraged seamlessly (eg, by clinical applications), behind the scenes, we enhanced a public open-source implementation of FHIR Genomics Operations.^1^^,^^31–33^ As shown in Figure 2, in the reference implementation, data (patient data, knowledge) are run through a normalization layer as part of ingestion. Likewise, upon receipt of a client API call, the genomic data server applies similar normalization to certain query parameters.

Because data are normalized upon ingestion, it becomes possible to ensure a consistent representation of genetic data in any Operation response. In the reference implementation, we return “raw” data and normalized data. For instance, where we ingest variant “NM_001195798.2: c.12G>A,” we normalize it to “NC_000019.9: g.11200236G>A,” and return both forms in a response. We find that the return of normalized data facilitates CDS rule writing, simplifying the number of constraints that need to be asserted.

### Variants

Various strategies for variant normalization in the FHIR Genomics Operations reference implementation have been tested over the past five years. Early experiments with normalization based on a synonym strategy using the ClinGen allele registry^34^ demonstrated feasibility, but did not address normalization of novel variants. Subsequently, the implementation shifted to the use of NCBI SPDI services, implemented as external API calls to the NCBI server. While NCBI SPDI services only normalize a subset of variants (eg, NCBI SPDI services do not currently process intronic variants such as NM_000038.5: c.1311_1312 + 1del), they are straightforward to call and allow for rapid development and experimentation. Based on our experience with NCBI SPDI services, we defined our variant canonical form as chromosome-level fully justified SPDI expressions.

To get around the need for calling external APIs (ie, the NCBI SPDI services), we are now moving to a new locally contained model, based on the biocommons/hgvs library.^8^ This package provides a python library to parse, format, validate, and normalize genetic variants. The package relies on three other libraries: biocommons/seqrepo (a repository of biological sequences); biocommons/uta (metadata for sequences in seqrepo, including transcript to chromosome alignment data); and biocommons/bioutils (common bioinformatics utilities, such as an HGVS to SPDI converter). The use of this library allows us to expand our canonical forms to be either canonical GRCh37 or GRCh38 SPDI or HGVS. (While we use canonical SPDI internally as our normal form, other systems may rely on, for instance, an HGVS normal form).

**Figure 3.**
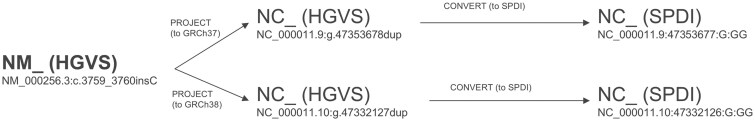
Pseudologic conversion starting with a transcript-level HGVS-formatted variant.

**Figure 4.**
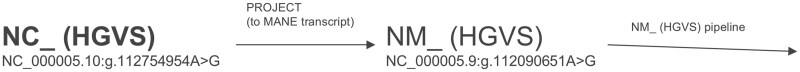
Pseudologic conversion starting with a chromosome-level HGVS-formatted variant.

**Figure 5.**
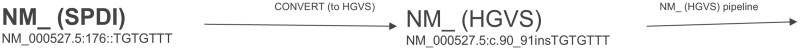
Pseudologic conversion starting with a transcript-level SPDI-formatted variant.

**Figure 6.**
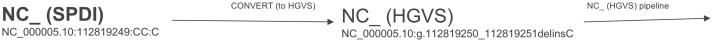
Pseudologic conversion starting with a chromosome-level SPDI-formatted variant.

The following Figures 3-6 show our high level pseudologic for normalizing variants submitted as query parameters, using biocommons/hgvs and associated biocommons packages. We focus primarily on logic that normalizes transcript and chromosome-level variants submitted in either HGVS or SPDI format, as these are the predominant formats being used in clinical scenarios today.

Figure 3 shows normalization logic for a transcript-level HGVS-formatted variant. Using positional information from the biocommons/uta database, we “project” the transcript onto the corresponding chromosome GRCh37 and GRCh38 sequences while also reformatting the chromosome-level variant to adhere to HGVS conventions. From there we convert to canonical SPDI format. Figure 4 shows normalization logic for a chromosome-level HGVS-formatted variant. Because the biocommons/hgvs library has no native function for liftover, we first project the variant onto a transcript (typically the MANE transcript^35^) so that we can then finish the normalization using the transcript-level HGVS-formatted variant pipeline from Figure 3. Likewise, Figures 5 and 6 show the normalization logic for SPDI-formatted variants. In both cases, the SPDI is first converted into HGVS, after which the HGVS pipeline is used.

### HLA alleles

Given the central authority of the IPD-IMGT/HLA Database, our team felt that any normalization solution would need to leverage this knowledge source. We found that the Python nmdp-bioinformatics/py-ard library is built and maintained by the NMDP, and easily enables a user of the library to refresh the underlying database as needed.

The py-ard library normalizes the ARD of an HLA allele into one of several normal forms. Because each normal form has pros and cons that can make a particular form more useful in a given scenario, our approach is to encapsulate the py-ard “lgx” normal form (two field ARD level representation), but also provide a utility that lets the client see other normal forms should they need them. We normalize ingested data and query parameters into py-ard “lgx” normal form. Pseudologic for search is:

- If search parameter is codeSystem and code, return patient records having an exact match.
- If search parameter is an arbitrary string
  - attempt to normalize string into py-ard “lgx” normal form.
  - return patient records where their HLA allele includes the string.
  - return patient records where their normalized HLA allele includes the normalized string.

### Annotations

For variant annotations, we focused on methods that address terminology variability related to conditions and medications. While we considered the use of an intermediary terminology normal form (eg, Ref.^36^-^38^), we ultimately determined that a method centered around terminology translations would be more scalable given that many high quality translations already exist, many of which are actively maintained.

We considered the terminology scenarios described above, recognizing that client needs vary, and concluded that no single encapsulation of terminology services would meet all user needs. As a result, for proof-of-concept testing, we limited our encapsulation to exact matches. In the exact match scenario, where a submitted SNOMED, ICD10CM, or RxNorm code has an exact match in the target terminology as recognized by a particular knowledge base, the submitted code is translated, and the target of the translation is then used to complete the query. For instance, if a client is querying using SNOMED's *Primary non-small cell lung cancer*, the encapsulation logic will automatically translate the code into Disease Ontology’s *Lung non-small cell carcinoma* when extracting annotations from CIViC. Note that our decision to limit proof-of-concept testing to exact matches was to enable a demonstration of terminology normalization encapsulation, and that a robust production system would need to consider a wider range of scenarios, such as pre- vs post-coordination and broader/narrower mappings.

Representative mappings were created for RxNorm to NCIt, and SNOMED and ICD10CM to MedGen and Disease Ontology. Mappings were limited to exact matches or, if there was no exact match, the closest broader concept in the target terminology (eg, SNOMED's *Lynch syndrome* is an exact match to MedGen’s *Lynch Syndrome* whereas SNOMED's *Fredrickson type IIa hyperlipoproteinemia* was mapped to the broader MedGen *Familial hypercholesterolemia*). In all cases, a given SNOMED, ICD10CM or RxNorm code is mapped to zero or one target code. Each map was converted into a FHIR ConceptMap and posted to a public FHIR server.

Pseudologic for search is:

- If submitted code is SNOMED, ICD10CM, or RxNorm
  - Retrieve relevant ConceptMaps from FHIR Server
  - Look for a code translation in the ConceptMap
    - If found, continue the query including the translated code
    - If not found, continue with submitted code

A high level summary of normalization encapsulation for variants, HLA alleles, and annotations is shown in Table 3.

**Table 3.** Summary of normalization strategy in FHIR Genomics Operations reference Implementation.

	Variants	HLA alleles	Annotations
**Libraries**	biocommons/hgvs; biocommons/uta; biocommons/seqrepo; biocommons/bioutils	nmdp-bioinformatics/py-ard	FHIR terminology services
**Canonical normal form**	Canonical SPDI	lgx reduction (reduce HLA allele to 2 field ARD level)	None (because here we are translating rather than normalizing)
**Normalization pseudologic**	Given a transcript or chromosomal variant, generate a GRCh37 and GRCh38 normalized HGVS and SPDI; Where normalization fails, attempt fallback using NCBI SPDI services.	Given an HLA allele, generate its lgx reduction.	Given a SNOMED, ICD10CM, or RxNorm code, attempt to map to a corresponding code in the target code system.
**Example**	**NM_001127510.3:c.145A>T** → { “b37SPDI”: “NC_000005.9:112102031:A:T”, “b38SPDI”: “NC_000005.10:112766334:A:T”, “b37HGVS”: “NC_000005.9:g.112102032A>T”, “b38HGVS”: “NC_000005.10:g.112766335A>T” }	DRB1*13:AKBAR → DRB1*13:02/DRB1*13:36/DRB1*13:67/DRB1*13:96/DRB1*13:109	http://snomed.info/sct **|126949007** → [ { “outcome”: “match found”, “code”: “DOID : 12689”, “system”: “https://disease-ontology.org/”, “display”: “Acoustic Neuroma” }, { “outcome”: “no match found”, “system”: “https://www.ncbi.nlm.nih.gov/medgen/” } ]

## Results

Algorithms for normalization of genetic variants and HLA alleles, and terminology translations, have been implemented and deployed in a public open source FHIR Genomics Operations reference implementation. All data and source code described in this report are located at https://github.com/FHIR/genomics-operations, and deployed at https://fhir-gen-ops.herokuapp.com/.

### Implementation demonstration

The following examples are live, and readers are welcome to explore them and/or test the implementation using their own examples. We demonstrate both “utility APIs” (utility APIs have not gone through an HL7 ballot, and are provided via the reference implementation) and FHIR Genomics Operations. For all examples, **baseURL = '**https://fhir-gen-ops.herokuapp.com’.

#### Variants

The “**normalize-variant**” endpoint is an operations utility API that demonstrates normalization of an arbitrary HGVS or SPDI-formatted variant into a canonical build 37 and build 38 HGVS and SPDI.

{baseURL}/utilities/normalize-variant? variant=NM_001127510.3: c.145A>T

returns:

{

 ”b37SPDI”: “NC_000005.9:112102031: A: T”,

 ”b38SPDI”: “NC_000005.10:112766334: A: T”,

 ”b37HGVS”: “NC_000005.9: g.112102032A>T”,

 ”b38HGVS”: “NC_000005.10: g.112766335A>T”

}

Variant normalization is encapsulated in all Operations that have a “variants” parameter, such as the “**find-subject-specific-variants**” operation. As a result, the following examples all return the same results.

See if patient m123 has a particular variant:

{baseURL}/subject-operations/genotype-operations/$find-subject-specific-variants? subject=m123&variants=**NM_001127510.3: c.145A>T**

{baseURL}/subject-operations/genotype-operations/$find-subject-specific-variants? subject=m123&variants=**NM_000038.6: c.145A>T**

{baseURL}/subject-operations/genotype-operations/$find-subject-specific-variants? subject=m123&variants=**NC_000005.10:112766334: A: T**

{baseURL}/subject-operations/genotype-operations/$find-subject-specific-variants? subject=m123&variants=**NC_000005.9: g.112102032A>T**

#### HLA alleles

The “**normalize-hla**” endpoint is an operations utility API that demonstrates normalization of an arbitrary HLA allele into various canonical forms.

{baseURL}/utilities/normalize-hla? allele=DRB1*13: AKBAR

returns:

{

 ”DRB1*13: AKBAR”: {

  ”G”: “DRB1*13:02:01G/DRB1*13:36/DRB1*13:67/DRB1*13:96/DRB1*13:109”,

  ”P”: “DRB1*13:02P/DRB1*13:36/DRB1*13:67/DRB1*13:96P/DRB1*13:109”,

  ”lg”: “DRB1*13:02g/DRB1*13:36g/DRB1*13:67g/DRB1*13:96g/DRB1*13:109g”,

  ”lgx”: “DRB1*13:02/DRB1*13:36/DRB1*13:67/DRB1*13:96/DRB1*13:109”,

  ”W”: “DRB1*13:02:01:01/DRB1*13:02:01:02/DRB1*13:02:01:03/DRB1*13:02:01:04/DRB1*13:02:01:05/DRB1*13:02:01:06/DRB1*13:02:01:07/DRB1*13:02:01:08/DRB1*13:02:01:09/DRB1*13:02:01:10/DRB1*13:02:01:11/DRB1*13:02:01:12/DRB1*13:02:01:13/DRB1*13:02:01:14/DRB1*13:02:02/DRB1*13:02:03/DRB1*13:02:04/DRB1*13:02:05/DRB1*13:02:06/DRB1*13:02:07/DRB1*13:02:08/DRB1*13:02:09/DRB1*13:02:10/DRB1*13:02:11/DRB1*13:02:12/DRB1*13:02:13/DRB1*13:02:14/DRB1*13:02:15/DRB1*13:02:16/DRB1*13:02:17/DRB1*13:02:18/DRB1*13:02:19/DRB1*13:02:20/DRB1*13:02:21/DRB1*13:02:22/DRB1*13:36/DRB1*13:67/DRB1*13:96:01/DRB1*13:96:02/DRB1*13:109”,

  ”exon”: “DRB1*13:02:01/DRB1*13:02:02/DRB1*13:02:03/DRB1*13:02:04/DRB1*13:02:05/DRB1*13:02:06/DRB1*13:02:07/DRB1*13:02:08/DRB1*13:02:09/DRB1*13:02:10/DRB1*13:02:11/DRB1*13:02:12/DRB1*13:02:13/DRB1*13:02:14/DRB1*13:02:15/DRB1*13:02:16/DRB1*13:02:17/DRB1*13:02:18/DRB1*13:02:19/DRB1*13:02:20/DRB1*13:02:21/DRB1*13:02:22/DRB1*13:36/DRB1*13:67/DRB1*13:96:01/DRB1*13:96:02/DRB1*13:109”,

  ”U2”: “DRB1*13:02/DRB1*13:36/DRB1*13:67/DRB1*13:96/DRB1*13:109”,

  ”S”: “DR6/DR13”

 }

}

HLA allele normalization is encapsulated in all Operations that have a “haplotypes” parameter, such as the “**find-population-specific-haplotypes**” operation. As a result, the following examples all find a patient whose HLA allele is reported as “DRB1*13: AKBAR.”

Find all patients having a particular HLA allele:

{baseURL}/population-operations/genotype-operations/$find-population-specific-haplotypes? haplotypes=DRB1*13: AKBAR&includePatientList=true

{baseURL}/population-operations/genotype-operations/$find-population-specific-haplotypes? haplotypes=DRB1*13:36&includePatientList=true

{baseURL}/population-operations/genotype-operations/$find-population-specific-haplotypes? haplotypes=DRB1*13:02:01:02&includePatientList=true

#### Annotations

The “**translate-terminology**” endpoint is an operations utility API that demonstrates terminology translations SNOMED to MedGen and DiseaseOntology; ICD10 to MedGen and DiseaseOntology; and RxNorm to NCIt.

{baseURL}/utilities/translate-terminology?codeSystem=http://snomed.info/sct&code=1259727001

returns:

[

 {

  ”outcome”: “match found”,

  ”system”: “https://disease-ontology.org/”,

  ”code”: “DOID : 3908”,

  ”display”: “Lung Non-small Cell Carcinoma”

 },

 {

  ”outcome”: “no match found”,

  ”system”: “https://www.ncbi.nlm.nih.gov/medgen/”

 }

]

Terminology translation is encapsulated in all Operations that have either a “conditions” or a “treatments” parameter, such as the “**find-subject-tx-implications**” operation. As a result, the following examples return the same results.

Find therapy options for patient CA12345 with non-small cell lung cancer having variant NM_001354609.2: c.1799T>A:

{baseURL}/subject-operations/phenotype-operations/$find-subject-tx-implications? subject=CA12345&variants=NM_001354609.2: c.1799T>A&conditions=https://disease-ontology.org**|DOID : 3908**

{baseURL}/subject-operations/phenotype-operations/$find-subject-tx-implications? subject=CA12345&variants=NM_001354609.2: c.1799T>A&conditions=http://snomed.info/sct**|1259727001**

### Lessons learned

Key lessons learned include: (1) integration of existing normalization software libraries into clinical search scenarios is feasible; (2) specific use cases guide the choice of normalization strategy; (3) normalization can fail.

While integration of normalization software libraries is feasible, integration is not “out of the box.” Many libraries (such as biocommons/hgvs) contain a wide range of capabilities, and only through use case analysis can an implementation define its own specific requirements. The design decisions described above may work well for a general purpose search strategy while falling short for a clinical application designed for, say, use by a transplant coordinator. The use of robust well-maintained libraries has not only been a time-saver, but more importantly, many of these libraries are very mature, addressing a number of edge cases in genomic data representation.

Every normalization strategy identified has limitations and categories of cases that fail normalization. Examples of normalization failures in our pipeline are shown in Table 4.

**Table 4.** Examples of normalization failures in our pipeline.

Example	Comments
**Variants**	
NG_012232.1(NM_004006.2):c.93 + 1G>T	Biocommons/hgvs does not support full HGVS syntax.
LRG_130:g.78815A>T	Our implementation currently supports normalization of variants expressed using NCBI transcript (“NM_”) and chromosome (“NC_”) reference sequences.
NM_001127510.1:c.145A>T	Biocommons/hgvs normalization requires that the transcript sequences and metadata be present in the biocommons/seqrepo and biocommons/uta databases, respectively.
**HLA Alleles**	
hla#3.33.0#HLA-A*01:03/HLA-A*03:01	Py-ard does not parse GL Strings and therefore will not normalize GL String components.
Bw4	Py-ard does not normalize epitopes.
HLA-A2 vs A2	Py-ard recognizes “A2” and not “HLA-A2” as a valid HLA serology representation.
LOINC 79712-6 (HLA-A*31:01 [Presence] in Blood or Tissue)	Our normalization pipeline does not currently extend to LOINC-encoded observations. For example, a search for “HLA-A*31:01” will not return an HL7 v2 observation with observation code of 79712-6.
**Annotations**	
NCIt C90565 | Buparlisib	Not all codes in target terminology are in source terminology. For example, experimental medications in NCIt may not be in RxNorm, and can therefore only be identified via an NCIt-based search (or basic string search).
SNOMED 1259754003 | Primary squamous non-small cell lung cancer	Our implementation currently supports a single best match, meaning that a query for SNOMED 1259754003 will identify knowledge coded with DOID : 3907 | Lung squamous cell carcinoma, but not knowledge coded with descendant DOID : 7045 | Basaloid lung carcinoma or ancestor DOID : 3908 | Lung non-small cell carcinoma.
Pre-coordination vs Post-coordination: SNOMED 372087000 | Primary malignant neoplasm **+** ICD-O-3 8070.3 | Squamous cell carcinoma	Our implementation currently supports a single best match and does not perform data normalization (eg, such as described in [36,39]). For instance, we do not attempt to compute the equivalence of this example with SNOMED 1259754003 | Primary squamous non-small cell lung cancer.

## Discussion

The FHIR Genomics Operations reference implementation has implemented a number of data normalization pipelines, which address many but not all potential data normalization scenarios. Major areas of ongoing work include defining strategies for enhanced retrieval of structural variants, “categorical variants,”^40^ and genomic findings that are not yet formally standardized in FHIR Genomics.

HGVS, GA4GH VRS, and FHIR Genomics do but SPDI does not provide a formalism for structural variants with imprecise endpoints. Our current normalization approach here is to project structural variants to their chromosomal coordinates so as to at least enable uniform chromosomal range-based search. A “categorical variant” is a variant class that potentially subsumes many different variants. For instance, a categorical variant “BRAF Class 2 Mutation” subsumes those BRAF variants associated with RAS-independent, constitutive kinase activity. Efforts within GA4GH^41^ and the FHIR community^42^ are advancing the ability to formalize categorical variants into canonical structures. For many clinically relevant genomic data types, such as fusions and translocations, formalizing the data exchange representation (eg, within the FHIR Genomics Reporting Implementation Guide) is a prerequisite for data normalization. Adding such data types into a balloted standard greatly constrains the universe of representations such that a data normalization pipeline then becomes feasible.

Interestingly, while the GA4GH VRS standard requires the inclusion of a canonical variant identifier, in practice, normalization is still required, if only to convert a variant in some other format into VRS. As such, the need for data normalization can be thought of as transcending any particular standard in that it is likely necessary for any implementation, whether based on FHIR or some other standard. Furthermore, data normalization is necessary whether considering targeted sequencing, whole exome sequencing, or whole genome sequencing.

## Conclusions

In this report, we have assessed data normalization considerations for genomic variants, HLA alleles, and variant annotations, and we have found that robust normalization algorithms can be encapsulated in clinical genomics APIs.

The notion of encapsulating genomic data normalization in search should not be limited to a FHIR Genomics Operations implementation. In fact, it is likely that similar capabilities would benefit many other types of genomic search interfaces.

We provide the reference implementation both as a community collaborative resource (also known as a playground for collaborative innovation) and also as a site where others can examine (and/or use) our solutions to help jump start their own implementations. Our intent is to continue to leverage the reference implementation as a valuable resource that reduces the burden of implementation in putting genomic medicine into practice.

## Data Availability

All data and source code described in this report are located at https://github.com/FHIR/genomics-operations, and deployed at https://fhir-gen-ops.herokuapp.com/.
